# New Clues to the Pathogenesis of Idiopathic Orbital Inflammation: Elevated IL‐8 and MCP‐1 in Tear Fluid

**DOI:** 10.1155/joph/4175012

**Published:** 2025-12-19

**Authors:** Jingqiao Chen, Wei Xiao, Huijing Ye, Zhihui Xu, Rongxin Chen, Huasheng Yang

**Affiliations:** ^1^ Department of Ophthalmology, The Second Affiliated Hospital of Soochow University, Suzhou, China, suda.edu.cn; ^2^ State Key Laboratory of Ophthalmology, Zhongshan Ophthalmic Center, Guangdong Provincial Key Laboratory of Ophthalmology and Visual Science, Sun Yat-sen University, Guangzhou, China, sysu.edu.cn

**Keywords:** biomarker, cytokine, idiopathic orbital inflammation, tear fluid

## Abstract

**Objectives:**

To characterize tear cytokine profiles in patients with idiopathic orbital inflammation (IOI) and analyze the expression of altered cytokines in blood and involved tissues.

**Methods:**

This case‐control study enrolled 18 IOI patients and 11 age‐/sex‐matched controls. Ocular Surface Disease Index (OSDI), corneal fluorescent staining, tear film breakup time (TBUT), Schirmer I test, and other clinical and laboratory parameters were obtained from all participants. Concentrations of cytokines in tear fluid, blood, and tissues were determined using a multiplex bead immunoassay system, enzyme‐linked immunosorbent assay, and immunohistochemistry.

**Results:**

Significantly elevated levels of interleukin (IL)‐8 and monocyte chemoattractant protein (MCP)‐1 (both *p* < 0.05) were found in IOI tear fluid. The area under receiver operating characteristic curve was 0.73 and 0.74 for IL‐8 and MCP‐1, respectively. IL‐8 and MCP‐1 were overexpressed in orbital tissues from patients with IOI, while the plasma levels of IL‐8 and MCP‐1 in the IOI group were parallel to the control group.

**Conclusions:**

The dysregulation of tear cytokine profiles provides a new insight into the potential immunologic mechanism for IOI. The elevated IL‐8 and MCP‐1 may represent candidate biomarkers of IOI.

## 1. Introduction

Idiopathic orbital inflammation (IOI), previously known as idiopathic orbital inflammatory pseudotumor (IOIP), is a fibroinflammatory disease characterized by inflammation of orbit and space‐occupying effects [1]. IOI can be classified into anterior, posterior, myositic, lacrimal, and diffuse based on the location of involvement [2]. Prompt diagnosis is vital to IOI management and prognosis; otherwise, untimely treatment may result in destructive fibrosis with blindness [3]. IOI is a diagnosis of exclusion, as the precise pathogenesis has not been verified. The refractory and recurrent nature of IOI suggests a complex molecular process, and the inflammatory microenvironment leading to immune system dysregulation constitutes the mainstay of IOI pathogenesis [4]. Previous study has identified localized cytokine dysregulation in orbital tissues [5]; however, the molecular mechanism and diagnostic strategy of IOI still deserve further exploration.

The diagnosis of IOI mainly relies on invasive procedures such as tissue biopsy [6]. Noninvasive markers, such as cytokines in tears, offer a promising alternative. Tear fluid is advantageous as a novel marker because of its ease of collection and potential to reflect local inflammation. Tear cytokines have been reported in orbital disease, such as thyroid‐associated ophthalmology (TAO) and extranodal marginal zone B‐cell lymphoma of ocular adnexa [7, 8]. Inflammatory involvement of the lacrimal gland may also contribute to ocular surface damage and increased inflammatory cytokines [7]. Therefore, we assume that altered cytokine profiles in tear fluid can be detected in IOI patients, which may contribute to the pathogenesis of the disease.

The pathophysiology of IOI is believed to involve a complex network of inflammatory mediators. Unraveling this network requires the simultaneous quantification of multiple cytokines. Multiple bead‐based immunoassays or high‐throughput assays have effectively addressed the limitation of quantifying multiple cytokines in trace tear samples. The value of this multiplexed approach has revolutionized the study of the ocular inflammatory milieu. It has been instrumental in delineating the cytokine profiles in various ophthalmic pathologies, such as dry eye disease [9], myopic macular degeneration [10], and glaucoma under chronic drug treatment [11], thereby providing unprecedented insights into disease mechanisms, potential therapeutic targets, and treatment responses.

In the present study, we evaluated tear composition in IOI patients and explored the expression of altered tear cytokines in the affected orbit and peripheral blood to identify potential biomarker candidates, which may add clues to the pathogenetic mechanism and pave the way for new treatment strategies for IOI.

## 2. Materials and Methods

### 2.1. Participant Enrollment and Specimen Collection

This study was conducted as per the Declaration of Tenets of Helsinki and approved by the Institutional Review Board of Zhongshan Ophthalmic Center. Written informed consent was obtained from all participants.

This preliminary cross‐sectional study comprised 18 patients with biopsy‐proven IOI who were treated at the Department of Orbital Disease and Ocular Oncology, Zhongshan Ophthalmic Center, from April 2019 to December 2019, and 11 age‐ and sex‐matched controls who did not have a history of inflammation or other ocular diseases. Exclusion criteria were patients with (1) other diseases that affect tear fluid, including dry eye, allergic conjunctivitis, lacrimal obstruction, glaucoma, uveitis, retinopathies, and TAO; (2) history of ophthalmic surgery in the past 6 months; (3) a history of glucocorticoids use within the past 3 months; (4) a history of eye drops other than artificial tears; (5) a history of contact lens wearing; and (6) pregnancy. In our study, diagnostic biopsy was obtained from patients with IOI with nonmyositic lesions as myositis is mostly a clinical and radiological diagnosis [12]. Therefore, orbital tissue was obtained in IOI patients with orbital fat or lacrimal gland involvement. The tear fluid and blood samples of the control group were obtained from age‐ and sex‐matched healthy volunteers. Normal orbital fat was obtained from subjects undergoing blepharoplasty, and normal lacrimal glands were collected from patients with lacrimal gland prolapse. Blood samples were obtained at the day before surgery.

Tear collection was completed prior to the ophthalmic examination. The tear fluid sample (10–50 μL) was collected in a sterile 20 μL capillary tube (Drummond Scientific, Broomall, PA, USA) before the surgery. All samples were collected between 10:00 AM and 16:00 PM, immediately expelled into sterile Eppendorf tubes, and stored at −80°C until being tested.

### 2.2. Clinical Examination

All individuals’ clinical data, including age, sex, and smoking history, were collected. Orbital magnetic resonance imaging (MRI) was performed in all patients with IOI to determine the involved site. Enrolled IOI patients and controls underwent ophthalmic examinations in the following order: Ocular Surface Disease Index (OSDI) questionnaire, tear collection, Schirmer I test, tear film breakup time (TBUT), and cornea fluorescent staining (CFS) [13]. The OSDI questionnaire was performed to quantify the subjective symptoms of the ocular surface [14]. The Schirmer I test was performed using sterile strips without anesthesia. TBUT was measured after corneal fluorescein sodium staining. The integrity of the tear film was examined by cobalt blue light, and the time before stained tear film defect appeared was recorded as TBUT. Subsequently, the entire cornea was examined and evaluated according to the Oxford Schema (I‐V) [13].

### 2.3. Multiplex Analyses of Cytokines

Cytokine concentrations in tear samples were blindly determined by a multiplex quantitative cytokine array (Milliplex Human Cytokine kit; Millipore Corp., Billerica, MA, U.S.A.) according to the manufacturer’s instructions. The following 23 selected cytokines were assayed: eosinophil chemotactic protein (eotaxin), fractalkine, IFN‐γ, growth‐regulated protein (GRO), interleukin (IL)‐10, monocyte chemoattractant protein (MCP)‐1, MCP‐3, IL‐12p40, macrophage‐derived chemokine (MDC), platelet‐derived growth factor AB/BB (PDGF‐AB/BB), IL‐17A, IL‐1β, IL‐2, IL‐4, IL‐5, IL‐8, IL‐13, interferon‐gamma‐induced protein 10 (IP‐10), macrophage inflammatory protein (MIP)‐1α, MIP‐1β, regulated on activation, normal T cell expressed and secreted (RANTES), soluble CD40 ligand (sCD40L), and tumor necrosis factor (TNF)‐α. The concentrations of cytokines in the tear samples were deduced from the standard curves and further corrected for the initial protein concentration.

Cytometric bead–based assay (CBA) was used to the measure the concentrations of IL‐8 and MCP‐1 in tear fluid according to the manufacturer’s protocol (BD Biosciences, San Jose, CA, USA) [15]. Briefly, 50 μL of tear sample was incubated with 50 μL of cytokine‐specific capture beads at room temperature for 1 h. Subsequently, 50 μL of phycoerythrin detection reagent was added to the tube and incubated again in the dark for 2 h. After incubation, 1 mL of wash buffer was added and centrifuged at 200 × *g* for 5 min. The supernatant was carefully removed by pipetting, followed by the addition of 300 μL wash buffer, and the samples were finally analyzed on a flow cytometer.

### 2.4. Immunohistochemistry (IHC)

The expression of IL‐8 and MCP‐1 was assessed by IHC. Formaldehyde‐fixed, paraffin‐embedded sections were deparaffinized and hydrated. Antigen was retrieved by using EDTA (pH 9.0) antigen retrieval solution. The sections were blocked at room temperature for 30 min, followed by incubation with primary antibodies against IL‐8 (Abcam, Cambridge, MA, USA)) and MCP‐1 (Servicebio, Wuhan, Hubei, China) at dilutions of 1:1000 and 1:500, respectively, overnight at 4°C. They were washed and incubated with secondary anti‐mouse and anti‐rabbit antibodies (Servicebio, Wuhan, Hubei, China) for 60 min. The reaction products were visualized with horseradish peroxidase as the enzyme and 3, 3‐diaminobenzidine (DAB).

### 2.5. Enzyme‐Linked Immunosorbent Assay (ELISA)

Plasma samples from IOI patients and controls were aliquoted and stored at −80°C until analysis. The concentrations of IL‐8 and MCP‐1 in the plasma were measured by specific ELISA kits, according to the instructions (R&D Systems, Systems, Minneapolis, MN, USA).

### 2.6. Statistical Analysis

Statistical analysis was performed using Prism (Version 8.0, GraphPad Software, Inc.) and SPSS (Version 22.0, IBM SPSS Statistics, Inc.). Categorical variables were determined using the chi‐square test or Fisher’s exact test. When the assumptions of normality and homogeneity of variance were satisfied, the means ± standard deviations (SDs) of normally distributed numerical variables were calculated and analyzed by unpaired *t*‐test. Otherwise, medians and interquartile ranges (IQRs) were calculated, and the quantitative variables were compared by using the Mann–Whitney *U* test. Spearman correlation analysis was performed to explore the relationships between cytokine levels in tear and blood. *p* < 0.05 was considered to indicate a statistically significant difference.

## 3. Results

### 3.1. Demographic and Clinical Data

A total of 18 patients with IOI and 11 control subjects were enrolled in the present study. The demographic and clinical parameters are shown in Table 1. There were no significant differences in age, sex, smoking history, TBUT, Schirmer I test, and CFS between the two groups (all *p* > 0.05). As expected, OSDI scores were higher in the IOI group than in the control group (*p* = 0.011). A total of 12 IOI patients had lacrimal gland involvement and 6 patients had orbital fat involvement.

**Table 1:** Clinical characteristics and demographic data of participants.

Characteristics	Control (*n* = 11)	IOI (*n* = 18)	*p*
Age (years) (mean ± SD)	44.64 ± 12.29	37.50 ± 12.98	0.154
Sex (male:female)	8:3	8:10	0.249
Smoking history [*n* (%)]	5 (45.5)	6 (33.33)	0.697
TBUT (s) (mean ± SD)	10.85 ± 1.66	9.01 ± 3.01	0.075
Schirmer I test (mm) (mean ± SD)	18.45 ± 6.44	16.72 ± 6.30	0.482
CFS (*n*, %)			
0	10 (90.91)	14 (77.78)	0.622
I	1 (9.09)	4 (22.22)	
II	0	0	
III	0	0	
IV	0	0	
V	0	0	
OSDI score [median (IQR)]	5.56 (0–17.50)	29.57 (4.45–42.86)	**0.011**
Lacrimal gland involved [*n* (%)]	—	12 (66.67)	‐

*Note:* Significant values of *p* (< 0.05) are in bold. IQR, interquartile range; TBUT, tear breakup time.

Abbreviations: CFS, corneal fluorescein staining; IOI, idiopathic orbital inflammation; OSDI, Ocular Surface Disease Index; SD, standard deviation.

### 3.2. Cytokines in the Tear Fluid From IOI Patients

Table S1 shows the effective range and detection rate of each cytokine. IL‐12p40, IL‐17A, IL‐2, and MIP‐1α did not reach the detection rate of 60% and no further analysis was performed. The concentrations of the remaining 19 cytokines in IOI group and control group are summarized in Table 2. Of all the 19 cytokines evaluated, only IL‐8 (*p* = 0.044) and MCP‐1 (*p* = 0.031) demonstrated a significant increase in tear fluid specimens from patients with IOI (Table 2 and Figure S1). However, there was no difference between IOI patients with and without lacrimal gland involvement (Figure S2).

**Table 2:** Concentrations of tear cytokines in IOI and healthy control groups.

Cytokines	Control median (IQR)	IOI median (IQR)	*p* ^†^
Eotaxin (pg/mL)	27.15 (11.46–94.52)	46.47 (12.50–86.75)	0.898
Fractalkine (pg/mL)	827.48 (657.36–1187.00)	1204.00 (893.51–2739.75)	0.102
IFN‐γ (pg/mL)	14.77 (2.35–140.17)	6.77 (2.35–105.69)	0.713
GRO (pg/mL)	3933.00 (3028.00–8035.00)	6217.00 (3803.25–14807.00)	0.148
IL‐10 (pg/mL)	7.17 (3.75–22.47)	10.11 (4.20–45.28)	0.667
MCP‐3 (pg/mL)	125.52 (82.03–211.39)	150.79 (50.22–354.46)	0.862
MDC (pg/mL)	391.62 (207.06–571.08)	414.90 (292.34–1335.50)	0.445
IL‐13 (pg/mL)	3.26 (1.46–7.40)	5.25 (2.16–23.00)	0.431
PDGF‐AB/BB (pg/mL)	107.75 (75.95–157.75)	166.27 (119.16–446.72)	0.094
sCD40L (pg/mL)	26.55 (8.90–61.07)	32.07 (11.71–202.30)	0.531
IL‐1β (pg/mL)	3.88 (2.20–11.83)	10.82 (5.23–31.60)	0.130
IL‐4 (pg/mL)	68.58 (28.17–176.06)	83.81 (12.84–546.08)	0.754
IL‐5 (pg/mL)	2.12 (1.23–4.92)	3.42 (1.69–10.32)	0.418
IL‐8 (pg/mL)	95.49 (69.89–386.29)	341.12 (124.28–974.62)	**0.044**
IP‐10 (pg/mL)	11,185.00 (7083.00–15160.00)	13,732.00 (8859.25–24985.25)	0.412
MCP‐1 (pg/mL)	375.83 (282.86–585.41)	813.42 (640.18–1624.25)	**0.031**
MIP‐1β (pg/mL)	22.46 (10.92–64.79)	70.64 (23.97–108.08)	0.204
RANTES (pg/mL)	34.41 (24.32–72.76)	63.70 (33.60–171.33)	0.261
TNF‐α (pg/mL)	5.33 (2.65–37.98)	8.86 (4.85–32.76)	0.686

*Note:* Significant values of *p* (< 0.05) are in bold. IgG4‐ROD, IgG4‐related ophthalmic disease; IQR, interquartile range; IFN = interferon; IL = interleukin; IP = interferon‐gamma‐induced protein.

Abbreviations: GRO = growth‐regulated protein, MCP = monocyte chemoattractant protein, MDC = macrophage‐derived chemokine, MIP = macrophage inflammatory protein, PDGF = platelet‐derived growth factor, and TNF = tumor necrosis factor.

^†^Mann–Whitney *U* test.

Next, we analyzed the receiver operating characteristic (ROC) curve to determine the potential value of IL‐8 and MCP‐1 in patients with IOI. IL‐8 (0.73) (Figure 1(a)) and MCP‐1 (0.74) (Figure 1(b)) had high areas under the receiver operating characteristic curves (AUROCs) to distinguish IOI from healthy controls.

Figure 1Receiver operating characteristic (ROC) curves for significantly changed tear cytokines in the diagnosis of IOI. The area under the ROC curve (AUROC) for IL‐8 (a) and MCP‐1 (b) from tear fluid.

**Figure:**
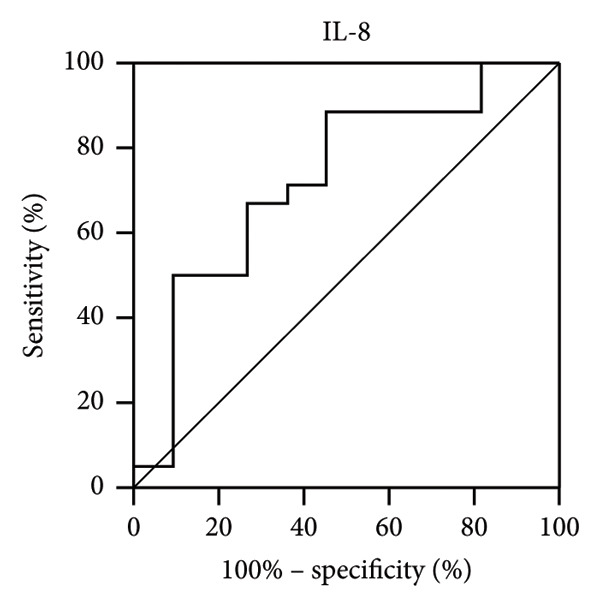
(a)

**Figure:**
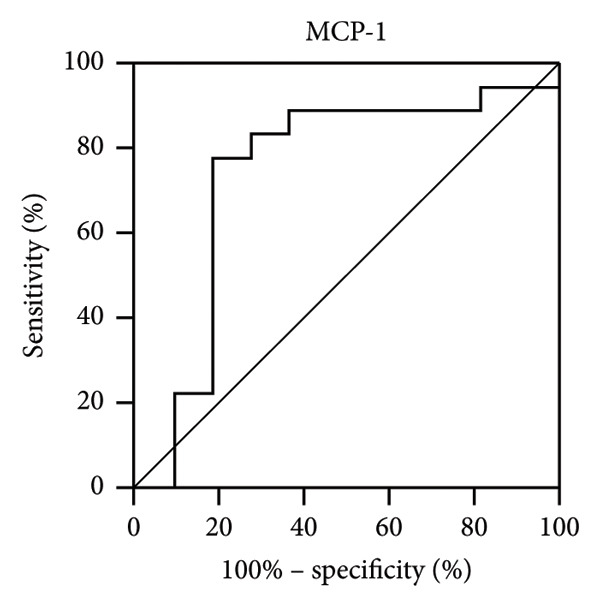
(b)

### 3.3. The Levels of IL‐8 and MCP‐1 in Blood and Orbit

To further clarify whether meaningful expressions of IL‐8 and MCP‐1 exhibit organ‐specific patterns, we examined their expression in peripheral blood and local tissues. As shown in Figure 2, the expressions of IL‐8 and MCP‐1 were significantly increased in affected orbital fat than that in the normal orbital fat, while slightly higher expressions of IL‐8 and MCP‐1 in inflamed lacrimal glands were observed compared to normal lacrimal glands. Nevertheless, there was no significant difference in the plasma level of IL‐8 between IOI patients and healthy subjects (*p* > 0.05) (Figure S3A), as well as MCP‐1 (*p* > 0.05) (Figure S3B). Furthermore, there was no association between plasma levels of IL‐8/MCP‐1 and their counterpart tear concentrations (Figure S3).

Figure 2Immunohistochemical staining for orbital tissues from IOI patients and control subjects. The expression of IL‐8 (a) and MCP‐1 (b) in orbital fat and lacrimal gland (original magnification, × 200 and × 400).

**Figure:**
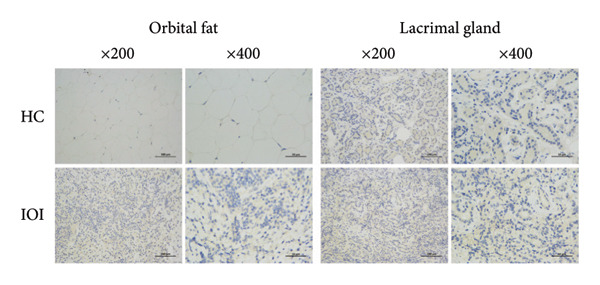
(a)

**Figure:**
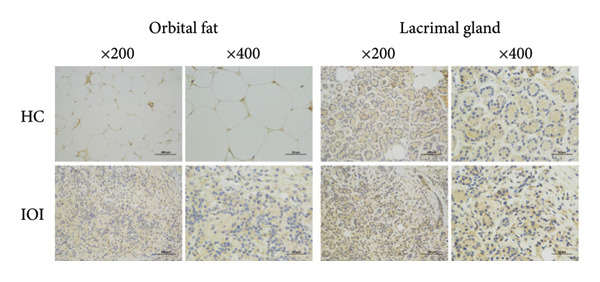
(b)

## 4. Discussion

To date, little is known about the pathogenesis of IOI. The infiltration of inflammatory cells and varying degrees of fibrosis constitute hallmark pathological characteristics of IOI [1, 16], prompting the hypothesis that this condition represents an immune‐mediated reaction to exposure from underlying pathogenic stimuli [6]. Previous studies have also suggested that IOI is characterized by increased proinflammatory cytokines in the inflamed orbit milieu [5]. However, the risk of performing a biopsy might outweighs the benefits, particularly in lesions involving the extraocular muscles, optic nerve, or orbital apex [4]. Recently, the cytokine antibody array–based tear analysis has been validated as a promising diagnostic biomarker for ocular pathologies and systemic disorders [17–21]. Therefore, tear‐based molecular biomarker is warranted and desirable in the application of IOI diagnosis. In this study, we tested 23 human cytokines in tear fluid from IOI patients and control subjects. IL‐8 and MCP‐1 in tear fluid from IOI patients were elevated. Moreover, similarly, IL‐8 and MCP‐1 were also increased in the affected orbital tissues. However, the corresponding cytokines in peripheral blood were comparable. These findings indicated that cytokine‐based inflammatory processes and immune dysregulation may be organ specific.

The lacrimal gland is the most frequent site of involvement in IOI and the major source of cytokines and chemokines in tears. Hence, tear analysis, which represents local ocular surface homeostasis, is still worth exploring. Our findings suggest that there may be no correlation between altered tear cytokine profiles and lacrimal gland involvement. However, Ang et al. have shown that tear inflammation cytokines may correlate with the pattern of orbital involvement. The expressions of tear cytokines were elevated in patients with orbital myositis complicated with dacryoadenitis [22]. Although there are differences between the two studies, the influence of lacrimal gland inflammation on IOI tears remains difficult to determine due to the lack of studies with large sample sizes in both.

Our results found that IOI leaves a molecular footprint in tear fluid with significantly higher levels of IL‐8 and MCP‐1. Chemokines are a group of cytokines that induce the directed migration of leukocytes, leading to the aggregation of migrating cells at the source of chemokine production. MCP‐1, known as CC‐chemokine ligand 2 (CCL2), is renowned for its function to attracting cells [23], including monocytes [24], T cells [25], and B cells [26], promoting inflammation and fibrosis progression [27, 28]. MCP‐1 has been identified as a key proinflammatory regulator of TAO pathogenesis [29]. IL‐8, also known as C‐X‐C motif chemokine ligand (CXCL8), is a proinflammatory chemokine produced by various cells to recruit granulocytes to sites of inflammation and promote the release of neutrophil extracellular traps (NETs) that kill invading microbes [30–32]. Combined with the increased expressions of MCP‐1 and IL‐8 in IOI patients, we theorized that MCP‐1 and IL‐8 may be involved in the pathogenesis of IOI and play a proinflammatory role.

The elevation of inflammatory mediators in tears raises the question of their upstream driving factors. Oxysterols, particularly 7‐ketocholesterol (7‐KC), have been increasingly recognized as potential contributors to ocular inflammation. Oxysterols, generated through cholesterol autoxidation, have been shown to induce oxidative stress and activate inflammatory signaling cascades in ocular tissues [33]. Notably, 7‐KC has been reported to promote NF‐κB activation and inflammasome assembly, leading to the secretion of proinflammatory mediators, such as IL‐8 and MCP‐1 [34–36]. These mechanisms may help explain the elevated levels of IL‐8 and MCP‐1 observed in the tear fluid of IOI patients. Strategies aimed at mitigating oxysterol‐induced inflammation may provide potential adjunctive therapeutic approaches for IOI.

In this study, we describe our finding that the high expressions of IL‐8 and MCP‐1 in tears from IOI patients were consistent with those in orbital tissues, whereas there were no significant differences in the expressions of plasma IL‐8 and MCP‐1 between the normal group and IOI group. This indicates that tear fluid, rather than peripheral blood, could reflect local orbital inflammation. The organ specificity of IOI may explain the inconsistency between the changes in orbit and peripheral blood.

To our knowledge, we present the first comprehensive analysis of tear cytokines in IOI patients, integrating multicompartmental profiling (tear, blood, and orbit) to identify disease‐specific biomarkers. One of the limitations is the relatively small sample size. This is a preliminary study, and larger sample size and prospective studies are needed to further verify the changes of tear cytokines in different disease states. Additionally, we did not collect reflex tear fluid with nasal stimulation, which shows the secretion capacity of the lacrimal glands. Besides the main and accessory lacrimal glands, the sources of cytokines and chemokines in tear fluid also include immunovigilant cells in the eye surface [37]. In our study, lacrimal gland involvement did not affect basal tear cytokine concentrations. IL‐8 and MCP‐1 were more significantly altered in the affected orbital fat than in the lacrimal gland, suggesting that the increase in tear cytokines and chemokines is more likely due to the chemotaxis of immunovigilant cells from the affected tissues to the ocular surface, rather than being a result of lacrimal gland secretion.

In conclusion, tear fluid is a novel area of IOI research and is more manageable, noninvasive, and easier to access. We have demonstrated that IOI patients exhibit higher expressions of IL‐8 and MCP‐1 in tears and orbital tissues. Hence, the discovery of tear‐derived biomarkers in IOI will enable their application in disease diagnostics and help elucidate the pathogenesis of IOI.

## Conflicts of Interest

The authors declare no conflicts of interest.

## Author Contributions

All authors contributed to the sample collection, data analysis, and interpretation, as well as the review and approval of the manuscript and the final decision to submit the manuscript for publication.

Jingqiao Chen, Wei Xiao, and Huijing Ye contributed equally to this work.

## Funding

This research was supported by the National Natural Science Foundation of China (nos. 82371099, 81700875, and 81870689), the Natural Science Foundation of Jiangsu Province for Youth (BK20230216), the Science and Technology Project of Suzhou (KJXW2022012), the Gusu Talent Program (GSWS2022050), and the Preliminary Research Project of the Second Affiliated Hospital of Soochow University (SDFEYJBS2109).

## Supporting Information

Additional supporting information can be found online in the Supporting Information section.

## Supporting information


**Supporting Information 1** Fig. S1: flow cytometric histograms of IL‐8 (A) and MCP‐1 (B) in tear fluid from IOI patients and healthy controls.

**Supporting Information 2** Fig. S2: the relationship between lacrimal gland involvement and cytokines from tear fluid. Comparisons of IL‐8 (A) and MCP‐1 (B) concentrations in tears between IOI patients with or without lacrimal gland involvement.

**Supporting Information 3** Fig. S3: cytokine concentrations in blood and their correlation with corresponding cytokines in tears. (A) The levels of IL‐8 in blood samples between IOI patients and healthy controls, and the correlation between tear IL‐8 and plasma IL‐8. (B) The concentration of plasma MCP‐1 between IOI patients and control subjects, and the correlation between tear MCP‐1 and plasma MCP‐1.

**Supporting Information 4** Table S1: detection rates of tear fluid by multiplex bead immunoassay.

## Data Availability

The data that support the findings of this study are available on request from the corresponding author. The data are not publicly available due to privacy or ethical restrictions.
