# Supplementary material for: New Clues to the Pathogenesis of Idiopathic Orbital Inflammation: Elevated IL‐8 and MCP‐1 in Tear Fluid
Source: J Ophthalmol. 2025 Dec 19;2025:4175012. doi: 10.1155/joph/4175012 (PMC12767073; doi:10.1155/joph/4175012)
Supplement: Supplementary file 1 — Supporting Information 1 Fig. S1: flow cytometric histograms of IL‐8 (A) and MCP‐1 (B) in tear fluid from IOI patients and healthy controls. [file JOPH-2025-4175012-s001.docx]

**Supplementary materials**


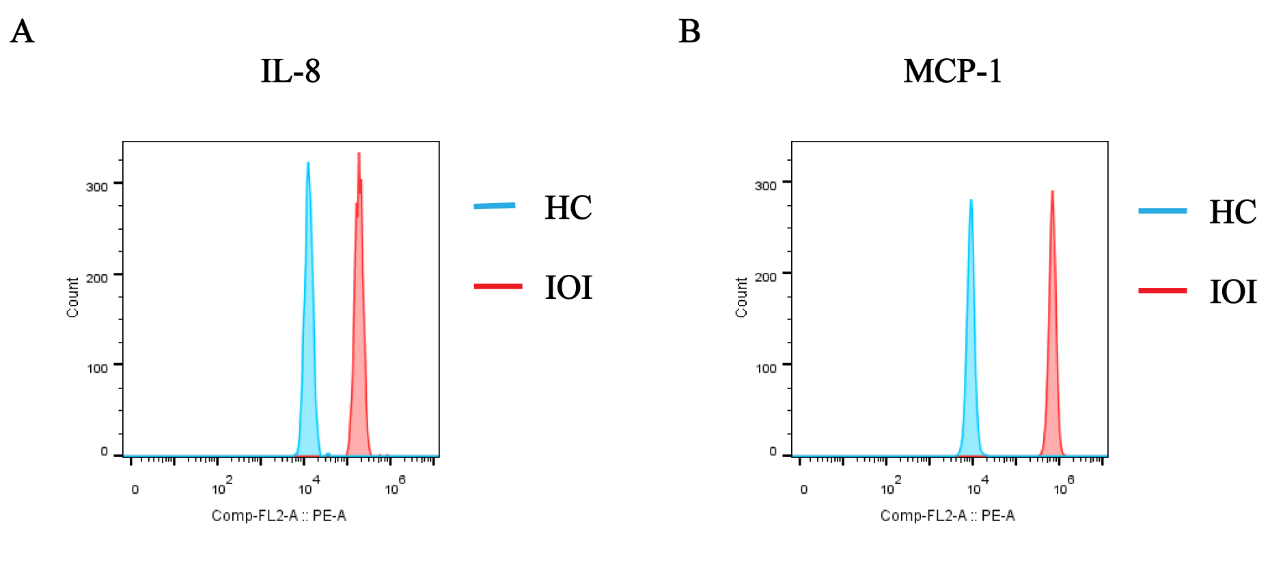


Fig. S1 Flow cytometric histograms of IL-8 (A) and MCP-1 (B) in tear fluid from IOI patients and healthy controls.
