# Supplementary material for: New Clues to the Pathogenesis of Idiopathic Orbital Inflammation: Elevated IL‐8 and MCP‐1 in Tear Fluid
Source: J Ophthalmol. 2025 Dec 19;2025:4175012. doi: 10.1155/joph/4175012 (PMC12767073; doi:10.1155/joph/4175012)
Supplement: Supplementary file 2 — Supporting Information 2 Fig. S2: the relationship between lacrimal gland involvement and cytokines from tear fluid. Comparisons of IL‐8 (A) and MCP‐1 (B) concentrations in tears between IOI patients with or without lacrimal gland involvement. [file JOPH-2025-4175012-s002.docx]

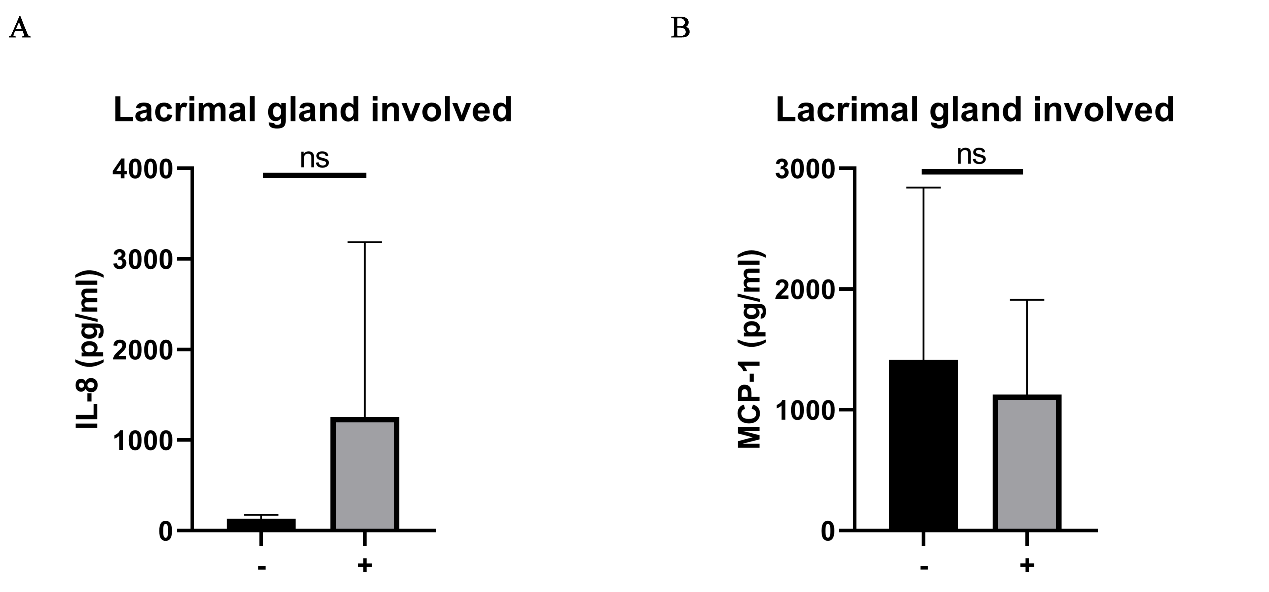
**Supplementary materials**

Fig. S2 The relationship between lacrimal gland involvement and cytokines from tear fluid. Comparisons of IL-8 (A) and MCP-1 (B) concentrations in tears between IOI patients with or without lacrimal gland involvement.
