# Supplementary material for: New Clues to the Pathogenesis of Idiopathic Orbital Inflammation: Elevated IL‐8 and MCP‐1 in Tear Fluid
Source: J Ophthalmol. 2025 Dec 19;2025:4175012. doi: 10.1155/joph/4175012 (PMC12767073; doi:10.1155/joph/4175012)
Supplement: Supplementary file 3 — Supporting Information 3 Fig. S3: cytokine concentrations in blood and their correlation with corresponding cytokines in tears. (A) The levels of IL‐8 in blood samples between IOI patients and healthy controls, and the correlation between tear IL‐8 and plasma IL‐8. (B) The concentration of plasma MCP‐1 between IOI patients and control subjects, and the correlation between tear MCP‐1 and plasma MCP‐1. [file JOPH-2025-4175012-s003.docx]

**Supplementary materials**


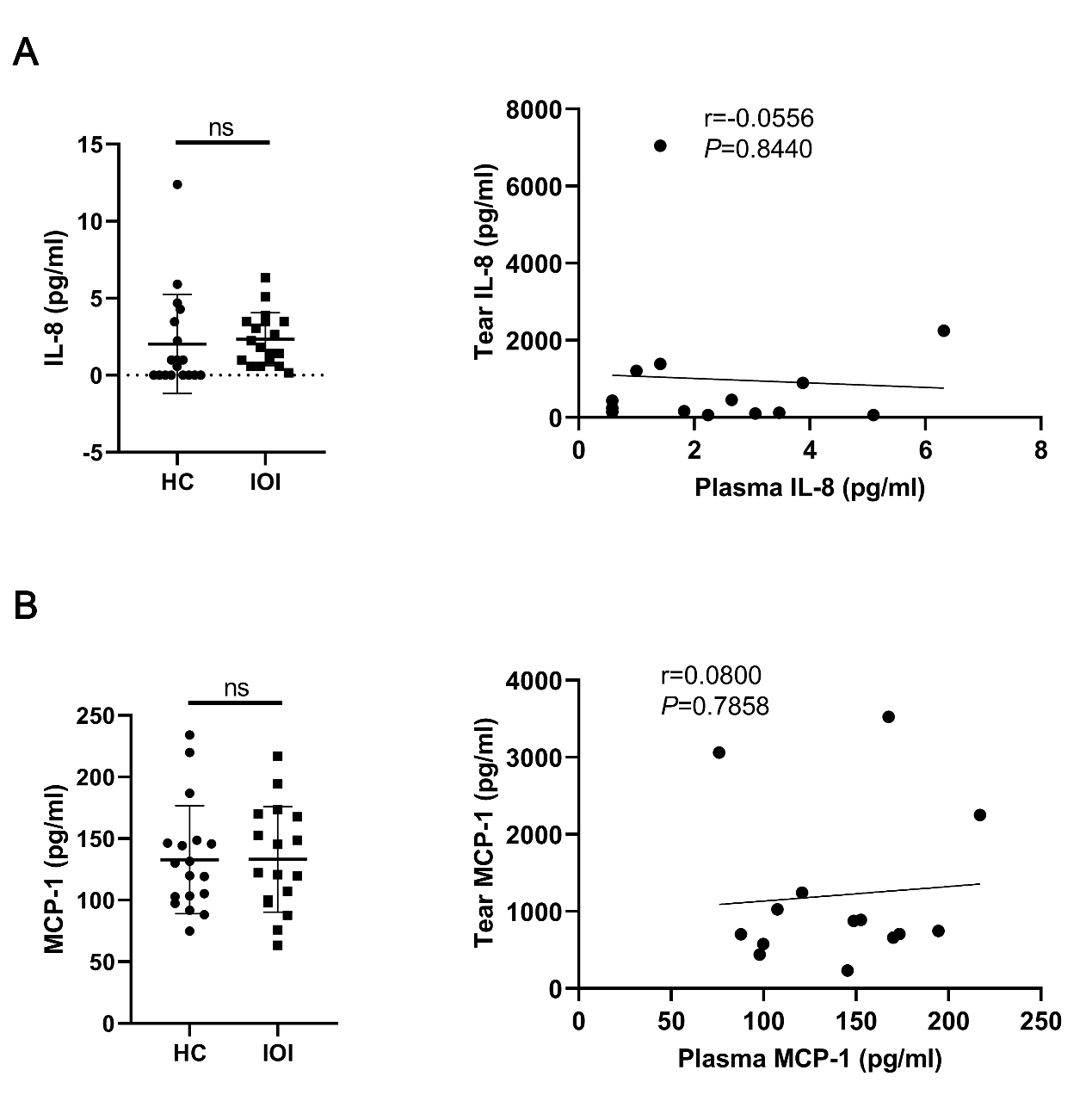
Fig. S3 Cytokine concentrations in blood and their correlation with corresponding cytokines in tears. (A) The levels of IL-8 in blood samples between IOI patients and healthy controls, and the correlation between tear IL-8 and plasma IL-8; (B) The concentration of plasma MCP-1 between IOI patients and control subjects, and the correlation between tear MCP-1 and plasma MCP-1.
