# Supplementary material for: New Clues to the Pathogenesis of Idiopathic Orbital Inflammation: Elevated IL‐8 and MCP‐1 in Tear Fluid
Source: J Ophthalmol. 2025 Dec 19;2025:4175012. doi: 10.1155/joph/4175012 (PMC12767073; doi:10.1155/joph/4175012)
Supplement: Supplementary file 4 — Supporting Information 4 Table S1: detection rates of tear fluid by multiplex bead immunoassay. [file JOPH-2025-4175012-s004.docx]

**Supplementary materials**

Table S1. Detection rates of tear fluid by multiplex bead immunoassay.

| Cytokines | Range | Control | |  | IOI | | *P*^†^ |
| --- | --- | --- | --- | --- | --- | --- | --- |
|  |  | n | % |  | n | % |  |
| Eotaxin | 1.22-44602 | 5/11 | 45.45 |  | 12/18 | 66.67 | 0.438 |
| Fractalkine | 1.90-21012 | 11/11 | 100 |  | 18/18 | 100 | NA |
| IFN-γ | 0.25-30592 | 6/11 | 54.54 |  | 11/18 | 61.11 | 1.000 |
| GRO | 4.54-20842 | 11/11 | 100 |  | 16/18 | 88.89 | 0.512 |
| IL-10 | 0.18-38322 | 11/11 | 100 |  | 18/18 | 100 | NA |
| MCP-3 | 1.32-19657 | 11/11 | 100 |  | 17/18 | 94.44 | 1.000 |
| IL-12p40 | 0.35-27412 | 3/11 | 27.27 |  | 8/18 | 44.44 | 0.449 |
| MDC | 2.63-19466 | 11/11 | 100 |  | 18/18 | 100 | NA |
| IL-13 | 0.10-62069 | 11/11 | 100 |  | 18/18 | 100 | NA |
| PDGF-AB/BB | 0.67-20960 | 11/11 | 100 |  | 18/18 | 100 | NA |
| sCD40L | 0.52-22802 | 11/11 | 100 |  | 15/18 | 83.33 | 0.268 |
| IL-17A | 0.75-19153 | 1/11 | 9.09 |  | 2/18 | 11.11 | 1.000 |
| IL-1β | 0.12-54331 | 11/11 | 100 |  | 15/18 | 83.33 | 0.268 |
| IL-2 | 0.12-53934 | 5/11 | 45.45 |  | 8/18 | 44.44 | 1.000 |
| IL-4 | 0.41-25194 | 11/11 | 100 |  | 17/18 | 94.44 | 1.000 |
| IL-5 | 0.11-57885 | 11/11 | 100 |  | 18/18 | 100 | NA |
| IL-8 | 0.10-63267 | 11/11 | 100 |  | 18/18 | 100 | NA |
| IP-10 | 1.02-20268 | 11/11 | 100 |  | 18/18 | 100 | NA |
| MCP-1 | 0.45-20012 | 11/11 | 100 |  | 18/18 | 100 | NA |
| MIP-1α | 0.67-11207 | 4/11 | 36.36 |  | 7/18 | 38.89 | 1.000 |
| MIP-1β | 0.41-18576 | 11/11 | 100 |  | 18/18 | 100 | NA |
| RANTES | 0.38-21179 | 11/11 | 100 |  | 18/18 | 100 | NA |
| TNF-α | 0.10-62871 | 11/11 | 100 |  | 17/18 | 94.44 | 1.000 |

IOI, Idiopathic orbital inflammatory; NA, not applicable. IFN = interferon; GRO = growth regulated protein; IL = interleukin; MCP = monocyte chemoattractant protein; MDC = macrophage-derived chemokine; PDGF = platelet-derived growth factor; IP = interferon gamma-induced protein; MIP = macrophage inflammatory protein; TNF = tumor necrosis factor.

^†^ Fisher’s exact test.
